# PGC-1alpha levels correlate with survival in patients with stage III NSCLC and may define a new biomarker to metabolism-targeted therapy

**DOI:** 10.1038/s41598-017-17009-6

**Published:** 2017-11-30

**Authors:** Alberto Cruz-Bermúdez, Ramiro J. Vicente-Blanco, Raquel Laza-Briviesca, Aránzazu García-Grande, Sara Laine-Menéndez, Lourdes Gutiérrez, Virginia Calvo, Atocha Romero, Paloma Martín-Acosta, José Miguel García, Mariano Provencio

**Affiliations:** 1 Servicio de Oncología Médica, Instituto de Investigación Sanitaria Puerta de Hierro-Segovia de Arana (IDIPHISA), Hospital Universitario Puerta de Hierro-Majadahonda, Madrid, Spain; 2 Flow Cytometry Core Facility, Instituto de Investigación Sanitaria Puerta de Hierro-Segovia de Arana (IDIPHISA), Hospital Universitario Puerta de Hierro-Majadahonda, Madrid, Spain; 3 Departamento de Bioquímica, Instituto de Investigaciones Biomédicas “Alberto Sols” UAM-CSIC and Facultad de Medicina, Universidad Autónoma de Madrid, Madrid, Spain; 4 Departamento de Patología, Instituto de Investigación Sanitaria Puerta de Hierro-Segovia de Arana (IDIPHISA), Hospital Universitario Puerta de Hierro-Majadahonda, Madrid, Spain

## Abstract

Lung cancer remains the leading cause of cancer-related death worldwide, with one-third diagnosed with locally advanced (stage III) disease. Preoperative induction chemo-radiotherapy is key for the treatment of these patients, however conventional cisplatin based approaches has apparently reached a plateau of effectiveness. In the search for new therapies, the targeting of tumor metabolism is revealed as an interesting option to improve the patient’s responses. Here we describe the importance of PGC-1alpha and GAPDH/MT-CO1 ratio levels as surrogates of the Warburg effect from a series of 28 stage III NSCLC patients, on PFS, OS and PET uptake. Moreover, our results show a great variability between tumors of different individuals, ranging from very glycolytic to more OXPHOS-dependent tumors, which compromises the success of therapies directed to metabolism. In this sense, using 3 different cell lines, we describe the relevance of Warburg effect on the response to metabolism-targeted therapies. Specifically, we show that the inhibitory effect of metformin on cell viability depends on cell’s dependence on the OXPHOS system. The results on cell lines, together with the results of PGC-1alpha and GAPDH/MT-CO1 as biomarkers on patient’s biopsies, would point out what type of patients would benefit more from the use of these drugs.

## Introduction

Lung cancer is the leading cause of cancer-related death worldwide, with an incidence around 19.4% of the total (1.2 million new cases per year). Histologically, we can differentiate two types: small-cell lung cancer and non-small cell lung cancer (NSCLC), being the latest the most common, counting for the 80–85% of the cases^1,2^.

Approximately one-third of patients with NSCLC are diagnosed with locally advanced (stage III) disease. Currently, the therapeutic strategy for these patients is the neo-adjuvant multimodal treatment, since local therapy alone (surgery or radiation) leads to poor overall survival because most of the patients die of distant metastases^3,4^. Thus, preoperative induction chemotherapy is key, because it can early eradicate distant micro-metastases and the down-staging increases the number of patients whose tumors are considered resectable, which contributes to improve survival^5^. After chemotherapy, surgery is potentially curative for these patients, being offered to the vast majority of patients with resectable N2 and to some patients with N3 node involvement (TNM staging system).

Sadly, conventional treatment of NSCLC has apparently reached a plateau of effectiveness in improving survival of patients, and treatment outcomes must still be considered disappointing^6^. Over the last few years, based on genetic alterations present in the tumor, new targeted therapies focused on the inhibition of key pathways for tumor proliferation have started to appear. However, these therapies do not cover most patients and have not yet reached the clinical routine for stage III patients. Thus, a need for new effective chemotherapy regimens that can be combined with local therapies (surgery and/or radiation) is still open^1,2,7^. In this sense, there is a considerable interest in identifying novel targeted agents that interfere with other hallmarks of NSCLC, such as tumor metabolism, which has recently aroused great interest.

The Warburg effect describes the preference of tumor tissues to obtain energy through glycolysis regardless of oxygen availability^8^. This predilection of glycolysis over the complete oxidation of nutrients via oxidative phosphorylation (OXPHOS) in the presence of oxygen (such as that performed by differentiated tissues), has to do with the facilitation to incorporate nutrients into biosynthetic pathways that support a rapid cell proliferation^9^.

PGC-1alpha is a transcriptional coactivator responsible for mitochondrial biogenesis. Thus in general terms, high levels of PGC-1alpha are associated with more OXPHOS-dependent metabolism of the cells, whereas low levels are associated with a more glycolytic phenotype^10–12^. However, despite its importance, the PGC-1alpha role in glucose metabolism reprograming in NSCLC has not been studied^13,14^.

Since the Warburg effect was described, many drugs targeting cancer metabolism have been developed. Unfortunately, there is a current unavailability on cancer drugs in the clinic targeting the tumor metabolism for lung cancer, despite the existence of scientific evidence *in vitro* and *in vivo* of its possible clinical utility^15,16^. In fact, several clinical trials based on this have been done or are currently ongoing, although their clinical benefits have not been as successful as one might expect^17^.

Remarkably, at the moment there are no good markers of response, most probably because mechanisms at the molecular level are still not clear. Thus, the characterization of these biomarkers is fundamental in order to identify patients who can benefit more from certain drugs targeting metabolism. This could explain why the clinical trials carried out have not been successful.

Therefore, knowing that the fundamental basis of treatment and possible cure of these patients is the neoadjuvant chemotherapy, we set out to identify the role of glucose metabolism in the stratification of patients at this stage, and in a second phase, to link these molecular findings with the sensitivity to different metabolic treatments.

## Results

### Prognostic value of Warburg Effect on stage IIIA NSCLC patients

To evaluate the importance of Warburg effect on NSCLC patients, we first analyzed the prognostic value of PGC-1alpha and GAPDH/MT-CO1 ratio mRNA levels on Overall-Survival (OS) and Progression Free Survival (PFS). In order to select a group of patients with similar characteristics, we focused on a cohort of 28 stage IIIA NSCLC patients. In this cohort the median PFS and OS were 18 [95% CI: 10.1–26.1] and 22.2 [95% CI: 8.9–35.4] months respectively.

PGC-1alpha is responsible for mitochondrial biogenesis. On the other hand, the GAPDH/MT-CO1 ratio is indicative of the balance between glycolysis and OXPHOS metabolism. GAPDH (glyceraldehyde phosphate dehydrogenase) is a key enzyme in glycolysis and MT-CO1 is one of the three mitochondrially encoded subunits that form the core of the Complex IV of the OXPHOS system.

**Figure 1:**
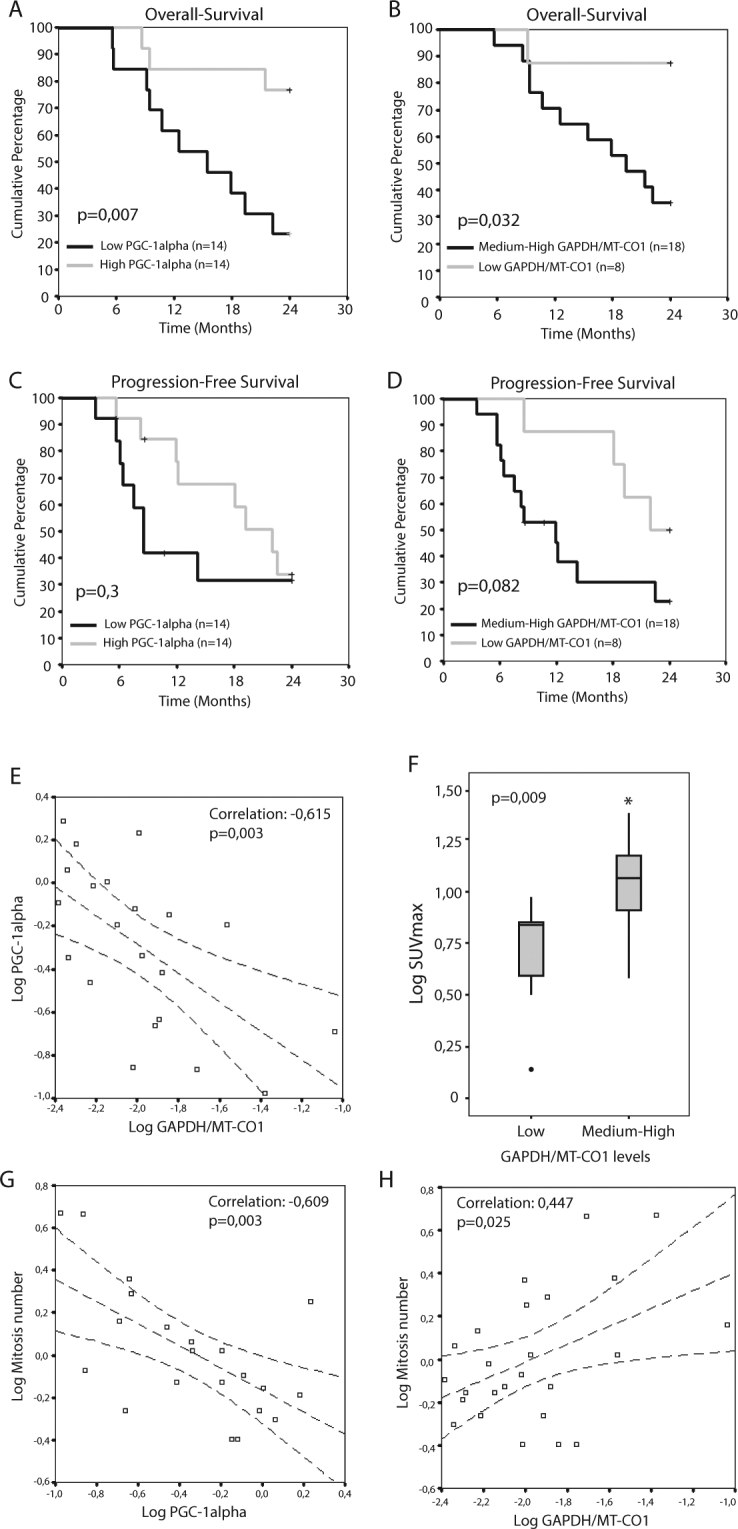
Warburg Effect on NSCLC patients. Overall-survival and Progression-Free Survival of NSCLC patients according to (**A** and **C**) PGC-1alpha or (**B** and **D**) GAPDH/MT-CO1 ratio mRNA levels. Low and High PGC-1alpha levels were defined by the median value of the population (14 patients in each group). Low and medium-High GAPDH/MT-CO1 ratio levels were defined by the tertiles (8 and 18 patients respectively). Note that a more glycolytic metabolism (Low PGC 1-alpha or medium-high GAPDH/MT-CO1 ratio) implies a worse prognosis. (**E**) PGC-1alpha mRNA levels inversely correlates to GAPDH/MT-CO1 ratio levels. (**F**) SUVmax levels in NSCLC patients in Low or Medium-High GAPDH/MT-CO1 groups. Relationship between tumor metabolism and the number of mitosis: **(G)** PGC-1alpha levels correlate inversely while (**H**) GAPDH/MT-CO1 ratio correlates directly with the number of mitosis. *P ≤ 0.05 was considered statistically significant.

Our results reveal a decreased OS for patients with PGC-1alpha low levels, median 15,4 months [95% CI: 7–23 months], compared to high levels patients with an OS superior to 24 months (Fig. 1A).

With respect to the GAPDH/MT-CO1 ratio, the patients were grouped into tertiles, grouping the medium and high levels in the same category since they behaved similarly compared to the low tertile. The median OS for low levels was higher than 24 months while for medium or high levels were 22.2 and 17.9 respectively (data not shown). Opposed to PGC-1alpha, low levels of the GAPDH/MT-CO1 ratio are indicative of a higher OS compared to the medium-high expression group: low ratio OS >24 months versus medium-high ratio 19.4 months [95% CI: 11.3–27.4 months] (Fig. 1B).

Although studies on PFS were not statistically significant, a clear difference was observed in the same sense as for OS, being high PGC-1alpha levels and low GAPDH/MT-CO1 ratio good prognostic factors. Comparing the PFS medians for both markers these differences are clearer: low PGC-1alpha 8,5 months [95% CI: 6.7–10.2] vs high PGC-1alpha 22 months [95% CI: 15.6–28.4], and medium-high GAPDH/MT-CO1 ratio 11,9 months [95% CI: 7.4–16.3] vs low GAPDH/MT-CO1 ratio 22 months. These obvious differences in median PFS are lost with the disease course, as the disease progresses in all patients (Fig. 1C–D).

These results clearly indicate that a more glycolytic metabolism (low PGC-1alpha or medium-high GAPDH/MT-CO1 ratio) confers a worse prognosis in both PFS and OS. Furthermore, the expression levels of both variables correlate inversely with a negative correlation index of 61% (Fig. 1E).

On the other hand, from the diagnostic positron-emission tomography (PET) images of the patients, the maximum standardized uptake value (SUVmax) levels of the tumor lesions were calculated. Due to the low number of PET images, the cohort was expanded to include other stages, thus obtaining a total of 22 patients with SUVmax information. The results show an increase in SUVmax levels in patients with medium-high levels of GAPDH/MT-CO1 (Fig. 1F).

Finally, to directly assess the role of the Warburg effect on the proliferative capacity of tumor cells, we analyzed the number of mitosis in hematoxylin-eosin tumor sections and correlated them with the expression levels of PGC-1alpha and GAPDH/MT-CO1 ratio as metabolism biomarkers. These data demonstrate an inverse correlation of the number of mitosis with PGC-1alpha levels (correlation of 61%) and a direct correlation with GAPDH/MT-CO1 ratio levels (correlation of 45%) (Fig. 1G–H).

### Warburg effect on NSCLC cell lines

**Figure 2:**
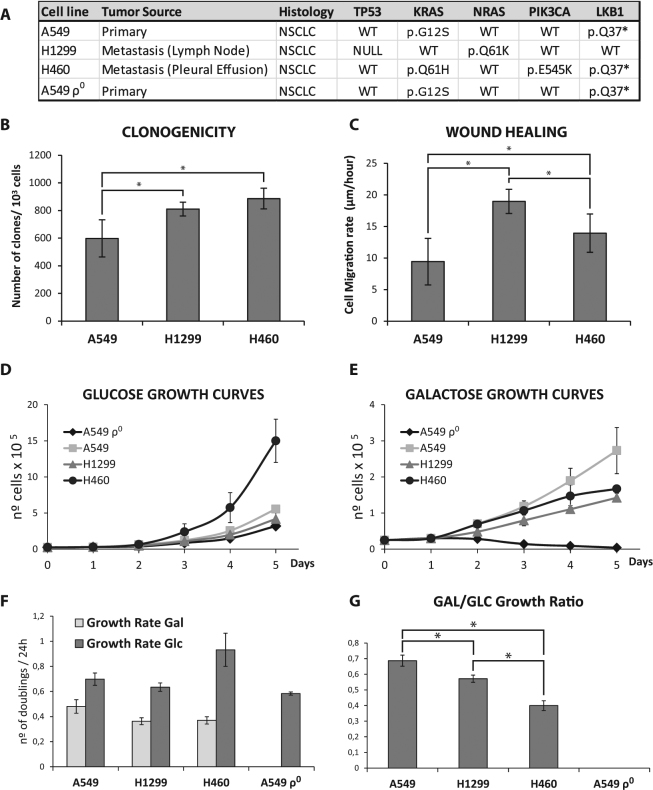
NSCLC cell lines. (**A**) Tumor source of the cell lines used in the study and their mutational status for TP53, KRAS, NRAS, PIK3CA and LKB1. (**B**) Clonogenicity assay, number of clones generated per 1000 cells seeded in DMEM after 14 days. (**C**) Wound healing assay. (**D**) Glucose (4,5 g/L) and (**E**) Galactose (0,9 g/L) growth curves for the cell lines. Note that the A549ρ^0^ are unable to growth in galactose media. (**F**) Growth rates (number of doublings per 24 hour) in glucose or galactose DMEM **(G)** Galactose to glucose growth rate ratios indicate the type of metabolism. In all cases, graphs represent the mean of three independent experiments and error bars indicate the standard deviation. ANOVA followed by Bonferroni post-test for multiple comparisons were used to analyze the differences in clonogenicity, migration, and galactose to glucose growth ratios. *P ≤ 0.05 was considered statistically significant.

To understand the molecular role of the Warburg effect in NSCLC, we decided to move on to an in vitro model using established cell lines. Three cell lines (A549, H1299, H460) from different origins and with different mutational profiles were chosen (Fig. 2A). The A549ρ^0^ cell line, derived from the A549 cell line, lacks the mitochondrial DNA (mtDNA) and serves as negative control of the OXPHOS function.

First, we set out to determine whether these cell lines have different aggressiveness as a consequence of their different origins: A549 primary tumor, H1299 lymph node metastasis and H460 pleural effusion. For that we characterized their migration capacity through wound healing assays and their clonogenicity. Our results showed an increased clonogenicity for H1299 and H460 cell lines compared to A549 cell line (Fig. 2B). Moreover, consistent with their origin, H1299 and H460 cells also showed higher motility than A549 cells. Additionally, H1299 cell line exhibited higher wound healing ability than H460 cell line (Fig. 2C).

Next, as a first approach to define their glucose metabolism, namely more glycolytic- or OXPHOS-orientated, we compared the cells abilities to grow in the presence of either glucose (Glc) or galactose (Gal) as carbon source (Fig. 2D–G). Our results showed significant differences in the Gal/Glc index among the three cell lines; having the A549 cells the highest Gal/Glc index, followed by H1299 cells and H460 consequently. Furthermore, as expected, the A549ρ^0^ line (without OXPHOS function) is unable to grow with galactose as sugar.

Then, to study further in-depth their metabolism and to verify that they behave in a similar way to the patients (more glycolysis, more aggressiveness), we analyzed different parameters such as oxygen consumption, reactive oxygen species (ROS) levels, glucose consumption and PGC-1alpha and GAPDH/MT-CO1 ratio levels.

**Figure 3:**
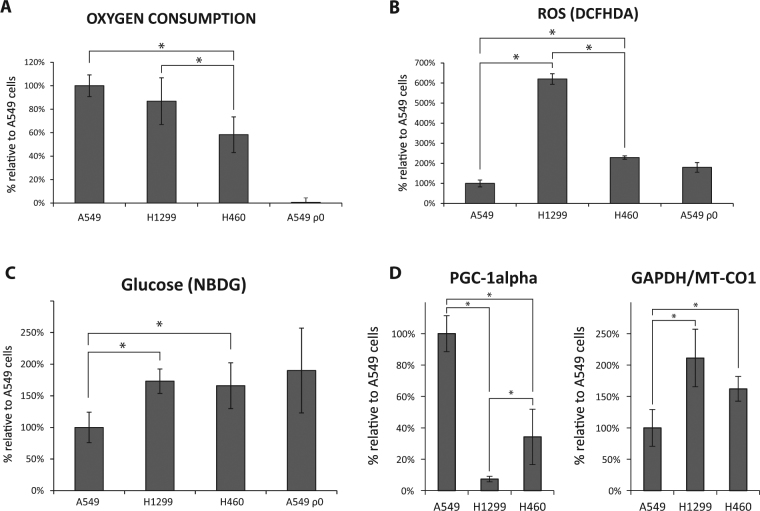
Warburg Effect on NSCLC cell lines. (**A**) Oxygen consumption of 4 × 10^6^ cells was recorded for 30 minutes using a Clark-type O_2_ electrode. (**B**) Total ROS levels were measured by flow cytometry with the fluorescent probe H_2_DCFDA. (**C**) Glucose consumption was evaluated with the 2-NBDG fluorescent probe by flow cytometry. (**D**) PGC-1alpha and GAPDH/MT-CO1 mRNA levels in the cell lines was evaluated by qRT-PCR using Taqman® gene expression assays. Graphs represent the mean of at least three independent experiments and error bars indicate the standard deviation. ANOVA followed by Bonferroni post-test for multiple comparisons were used to analyze the differences in the parameters. *P ≤ 0.05 was considered statistically significant. A549ρ^0^ were excluded from the statistical analyses to facilitate the graph interpretation.

Consistent with previous findings, the oxygen consumption rate showed a similar behavior to the Gal/Glc ratio, with significant differences between the H460 and A549 or H1299 cell lines. Despite the decrease in oxygen consumption of H1299 cells compared to the A549 cells, this difference does not become statistically significant (Fig. 3A).

ROS levels are associated with defects in the OXPHOS function and with a higher degree of tumor progression. Our results indicate that H1299 has the highest ROS levels among the cell lines, followed by H460 cells which have higher ROS levels than A549 cells (Fig. 3B).

Glucose consumption was assessed with the 2-NBDG fluorescent probe, similar to the fluorodeoxyglucose (FDG) used in patient’s PET images. We observed a significant increase in glucose consumption for the H1299 and H460 cell lines with a more glycolytic profile, although no differences were observed between them (Fig. 3C).

Finally, PGC-1alpha and GAPDH/MT-CO1 levels were evaluated (Fig. 3D). A549 cells presented the highest PGC-1alpha levels, followed in second place by the H460 cells. H1299 cells showed lower levels of PGC-1alpha than A549 or H460 cells. On the contrary, GAPDH/MT-CO1 is expressed inversely to PGC-1alpha in the cell lines; GAPDH/MT-CO1 ratio level was lower in A549 than in H460 and H1299 cell lines, although no significant differences were observed between these two cell lines.

### Targeting of Warburg effect on NSCLC cell lines

Once the cell lines were metabolically characterized and verified that the relationships between metabolism and aggressiveness parameters were similar to those found in patients, we aimed, as a proof of concept, to test their sensitivities to two drugs (2-deoxy-glucose and metformin) targeting different types of metabolism.

**Figure 4:**
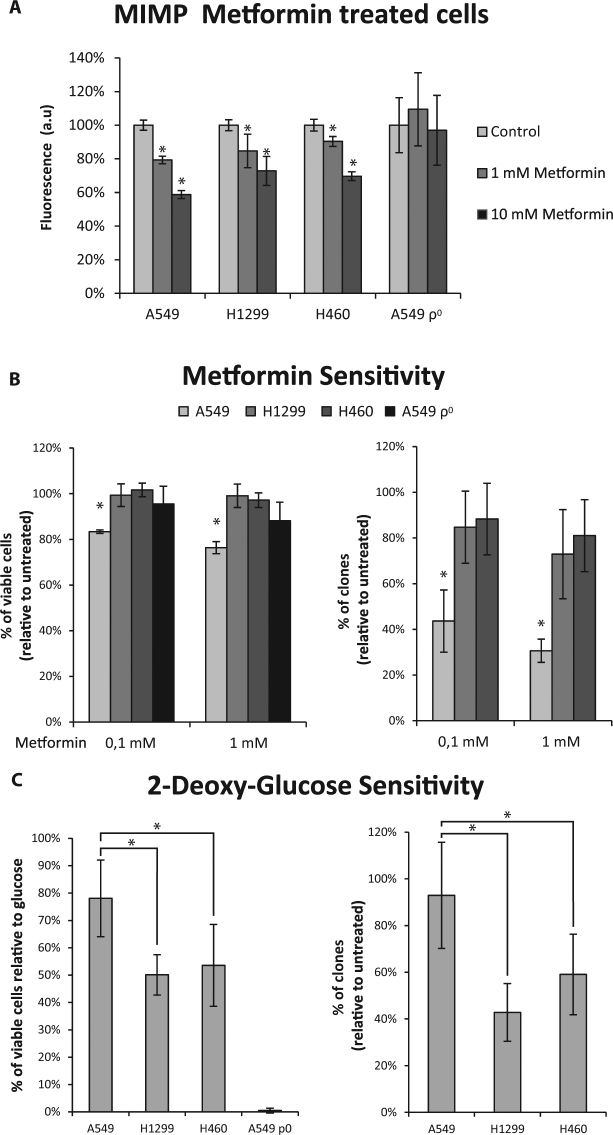
Targeting of Warburg effect on NSCLC cell lines. (**A**) Metformin treatment reduces the mitochondrial inner membrane potential (MIMP). Note that the MIMP of A549ρ^0^ cells is not modified by metformin treatment (**B**) Metformin sensitivity among cell lines correlates to OXPHOS dependence. (**C**) 2-deoxy-glucose sensitivity among cell lines correlates to glycolysis dependence. 2-tailed Student’s t test was used to analyze differences in MIMP and cell viability after metformin treatment for all the cell lines. *P ≤ 0.05 was considered statistically significant.

It has been reported that metformin acts as an inhibitor of Complex I of the OXPHOS system. To check the effect of metformin on the OXPHOS function, we treated all cell lines with 1 mM or 10 mM metformin for 24 hours (Fig. 4A).

Our results showed a significant decrease in mitochondrial inner membrane potential (MIMP) for all cell lines dependent on the concentration of metformin present. The only cell line that did not present this effect was the A549ρ^0^, whose MIMP remained constant with the treatments.

Then, we evaluated the sensitivity of the cell lines at low doses of metformin (0.1 and 1 mM), showing a decrease in the number of viable cells for A549 cell line for both doses in a concentration dependent way. On the contrary, it appears that cell lines H1299, H460 and A549ρ^0^ are not sensitive to the effect of metformin at low doses since there are no significant differences compared to untreated cells. Consistent with this result, the clonogenicity assays showed greater metformin sensitivity for A549 than for H1299 and H460 cell lines, which exhibited reduced metformin sensitivity (Fig. 4B).

On the other hand, we evaluated the sensitivity of the cell lines to 2-deoxy-glucose treatment. 2-deoxy-glucose is an analogous derivative of glucose that is not capable of being metabolized by hexokinase, thus blocking glycolysis. Sensitivity to 2-deoxy-glucose exhibits an opposite behavior to metformin sensitivity. The A549ρ^0^ cell line is extremely sensitive (there are no viable cells after treatment), followed by H1299 and H460 cell lines which reduce their viability to approximately 50%. Finally, the A549 cell line is the least sensitive, reducing its viability only 20% compared to untreated cells. In the same way, the number of colonies after 2-deoxy-glucose exposure is lower for H1299 and H460 compared to A549 cells (Fig. 4C).

## Discussion

The role of tumor metabolism has acquired relevance in recent years as one of the hallmarks of cancer. Despite this, there are no therapies in the clinical routine based on the targeting of this altered metabolism. However, there are currently ongoing clinical trials with different drugs targeting metabolism. In any case, there are no predictive markers of response to these type of therapies, which compromises the success of many drugs to improve the standard of care due to the inter-individual metabolic heterogeneity of tumors. In our study, we combined both patients and *in vitro* analysis and proposed some treatment response markers to metabolism targeted therapies, based on tumor glucose utilization phenotype and not on mutational profile of key driver genes. Interestingly, our data suggest a potential association between PGC-1alpha mRNA levels and metabolic phenotype in patients’ tumor biopsies.

Our data in patients show that there is a great heterogeneity between tumors of different individuals, having very glycolytic tumors (High SUVmax, High GAPDH/MT-CO1 ratio, Low PGC-1alpha) and tumors with an energetic profile more based on their OXPHOS system (Low SUVmax, low GAPDH/MT-CO1 ratio, High PGC-1alpha levels). In addition, our results confirm that the Warburg effect has a clinical relevance in NSCLC, both in PFS and OS. The majority of the studies in stage IIIA, even with the heterogeneity of these patients, show a PFS and OS in the order of months, which vary in function of diverse prognostic factors. In our cohort of patients the median OS was 22 months similar to other stage IIIA patient cohorts^18^. In our study, patients with high levels of PGC-1alpha or low GAPDH/MT-CO1 ratio had a median OS higher than 24 months, compared to those with low PGC-1alpha expression or high GAPDH/MT-CO1 ratio, who had a median OS of 15.4 or 19.4 months respectively. In a similar way, patients with high PGC-1alpha or low GAPDH/MT-CO1 ratio had a median PFS of 22 months versus those with low PGC-1alpha expression or high expression of GAPDH/MT-CO1 ratio who had median PFS of 8.5 or 11.9 months respectively.

Thereby, our results demonstrate that the glycolytic phenotype correlates with a worse prognosis in both PFS and OS in stage IIIA NSCLC patients. Similar conclusions for stage I NSCLC patients were obtained from the proteomic-based bioenergetic cellular index^19^. The inverse relationship of PGC-1alpha and GAPDH/MT-CO1 ratio with PFS and OS of patients coincides with the function of these genes related to glucose metabolism and with the Warburg effect. Indeed, both variables correlate inversely, reinforcing this affirmation.

Next, due to the impossibility of directly asses drug response in patients, we performed *in vitro* studies with cellular models to find markers that define sensitivity to drugs directed to glucose metabolism.

Prior to determine their drug sensitivity, we characterized their aggressiveness and biochemical function. Similar to the results in patients, our cell lines with different aggressiveness showed different metabolisms. A549 cell line showed the weakest malignant phenotype, with low clonogenic potential, migration capacity and a growth rate. On the other hand, H1299 and H460 showed higher aggressiveness than A549 cell line for most of the analyzed parameters. Regarding to metabolism characterization, A549 cell line showed a more OXPHOS metabolism, with higher Gal/Glc index, greater oxygen consumption, lower glucose consumption, elevated levels of PGC-1alpha and lower levels of GAPDH/MT-CO1, whereas the tumor cell lines H1299 and H460 turned out to be more glycolytic, with lower Gal/Glc index, lower oxygen consumption, greater consumption of glucose, reduced levels of PGC-1alpha and elevated GAPDH/MT-CO1 ratio. Nevertheless, there are differences between H1299 and H460 cell lines for some of these parameters although not enough clear to observe a pattern, which make it impossible to conclude differences between these two cell lines. However, the relationship between higher glycolysis with increased malignancy described in our cell lines is similar to other tumor types^20^.

Finally, we measured cell lines’ sensitivity to metformin and 2-deoxy-glucose. We propose that metformin affects the cells more when they are more OXPHOS-dependent. Recently, it has been described that metformin is an inhibitor of the complex I^21^. In our study we demonstrate that this compound reduces the MIMP of cells, most probably as a consequence of Complex I inhibition, since Complex I is mainly responsible for MIMP maintenance. Thus, treatment with metformin does not reduce MIMP in A549ρ^0^ cells, since it is maintained by another mechanism and not through Complex I activity^22^. Furthermore, when A549 cell line (the one with higher OXPHOS function and highest metformin sensitivity) lacks the mtDNA and therefore is forced to lose its OXPHOS dependence, is no longer sensitive to metformin. On the other hand, H1299 and H460 cell viability is not severely affected, even though metformin decreases their MIMP. This is probably because their MIMP and the associated OXPHOS function are not so important for their growth, as demonstrated by their biochemical parameters. In contrast, 2-deoxy-glucose, which inhibits hexokinase, and blocks the glycolytic pathway, affects the cell lines that are more glycolysis-dependent, such as H1299, H460 and A549ρ^0^ but does not disturb the more OXPHOS-dependent A549 cell line.

Our study point out, through PGC-1alpha levels, an easy manner of assessing the metabolic phenotype in patients and also suggest a correlation with sensitivity to metabolism targeted drugs. The approach proposed, focused more on the phenotype than on the tumor genotype, avoids the difficult interpretation of complex genomic results, even more for a phenotype that can be regulated by multiple genes, as well as, by epigenetic and microenvironmental factors. Further studies will clarify the relationship of these factors in lung cancer, defining the tumor metabolism in a more comprehensive fashion. This knowledge could have implications for the patients’ diagnosis, follow-up and treatment.

## Conclusions

To summarize, our results indicate that exist variability in glucose metabolism pathways in lung cancer patients’ tumors and established cell lines. In this respect, PGC-1alpha mRNA levels inversely correlate with GAPDH/MT-CO1 ratio. Interestingly, both markers have a clinical relevance in prognosis (PFS and OS), supporting the relationship between a more glycolytic metabolism with a more aggressive phenotype. Furthermore, the *in vitro* drug-sensitivity in cell lines would suggest that PGC-1alpha and GAPDH/MT-CO1 levels could be used to predict patient response to treatments based on targeting glucose metabolism on lung cancer.

## Methods

### Patients

All the experiments carried out in this study complied with current Spanish and European Union laws and the principles outlined in the Declaration of Helsinki. The study and experimental protocols were approved by the Hospital Universitario Puerta de Hierro Ethics Committee and written informed consent was obtained for all the patients recruited.

A cohort of 28 stage IIIA NSCLC patients was studied. Clinical data from these patients: Overall-survival (OS), Progression-Free survival (PFS), SUVmax levels, etc. as well as molecular data from diagnostic biopsies, were obtained for the analysis.

Unfortunately we were only able to obtain PET images for 8 of these patients. Thus, the cohort was increased for SUVmax correlations, retrieving the SUVmax levels from another 17 patients to a total number of 25 patients with SUVmax data (I = 6, II = 6, IIIA = 8, IIIB = 4 IV = 1).

### Mitotic count

The number of mitotic figures was evaluated in the hematoxylin-eosin diagnostic slides using high-power field (HPF) at × 400 magnification^23^. Per sample, 10 HPF were analyzed by two independent anatomical pathology technicians and the mean was calculated.

### Cell Lines

A549, H1299 and H460 were obtained from the ATCC. A549 ρ^0^ cells were kindly provided by Dr. Rafael Garesse, from UAM (Madrid). All cells were cultured routinely in DMEM, with 10% Fetal Bovine Serum (FBS), penicillin streptomycin (Gibco) and uridine (Sigma). The mutational landscape of TP53, KRAS, NRAS, PIK3CA, LKB1 was retrieved from COSMIC database.

### Wound healing assay

Cells were cultured in a 24-well plate until they reached 90% confluence and then scratched with a 200 µL pipette tip. The wounded area was photographed at the start (t = 0 h) and at time point (t = 16 h). Pictures were analyzed using Image J software and the migration rate was calculated as the distance recovered by the migration front per time unit (µm/h). Assays were performed in at least 3 independent experiments.

### Expression analysis

The RNA from cells was extracted using the “RNeasy mini Kit with DNAse” (Qiagen). The RNA extraction from FFPET biopsies was performed using the “High Pure FFPET RNA isolation Kit” (Roche). cDNA was synthetized using the “NZY First-Strand cDNA Synthesis Kit” (NZYtech).

mRNA expression was evaluated by qRT-PCR using the following Taqman® gene expression assays: PGC-1alpha(Hs01016719_m1), GAPDH (Hs02758991_g1), MT-CO1 (Hs02596864_g1). TBP (Hs00427621_m1) was used as endogenous control^24^.

### Glucose and galactose growth curves

Cell growth was evaluated after seeding 6 well plates at a density of 25,000 cells/well and growing the cells for 4 days in DMEM containing either 4.5 g/L glucose (Glc) or 0.9 g/L galactose (Gal). The cells were harvested and counted every 24 hours. Population doubling times were calculated using *Doubling Time Software v1*.0.10 (http://www.doubling-time.com). Assays were performed by duplicate in at least 3 independent experiments.

### Metformin sensitivity assays

3 × 10^3^ cells per well of a 96-well plate were cultured in DMEM overnight. The next morning the medium was replaced for DMEM containing the different metformin treatments according to figure legends and incubated for 48 hours. Cell viability was assayed using the Cell Counting Kit-8 (CCK-8) (Dojindo EU GmbH, Munich, Germany) according to the manufacturer’s protocol. Metformin (Sigma) was diluted to 1 M stock, aliquoted and frozen until its use. Assays were performed by duplicate in at least 3 independent experiments.

### 2-deoxy-Glucose sensitivity assays

6 × 10^3^ cells per well of a 96-well plate were cultured in DMEM overnight. The next morning the medium was replaced for a solution (156 mM NaCl, 3 mM KCl, 2 mM MgSO_4_, 1.25 mM KH_2_PO_4_, 2 mM CaCl_2_, 20 mM HEPES, and 5 mM pyruvate pH 7.35) containing 5 mM 2-deoxy-D-glucose or 10 mM glucose^25^. After 6 hours of incubation the percentage of viable cells in 2-d-G relative to glucose was evaluated using the Cell Counting Kit-8 (CCK-8) (Dojindo EU GmbH, Munich, Germany) according to the manufacturer’s protocol. Assays were performed by duplicate in at least 3 independent experiments.

### Clonogenicity assays

50.000 cells per well were seeded on 6-well plates. The next day medium was replaced for DMEM containing the different metformin or 2-d-G treatments. After 72 h incubation cells were trypsinized and 1,000 single viable cells were plated in 100-mm Petri dishes. The cells were then grown for 14 days and the number of colonies counted using 2% crystal violet. Assays were performed in at least 3 independent experiments.

### Oxygen consumption

The basal respiration of 4 × 10^6^ intact cells was measured at 37 °C using a Clark-type oxygen electrode (Hansatech Instruments) as described^26^. The mean of at least three independent experiments is shown.

### Flow cytometry

Cytoplasmic ROS were assessed using 2′,7′-dichlorodihydrofluorescein diacetate (H_2_DCFDA, Invitrogen). The mitochondrial inner membrane potential (MIMP) was evaluated using tetramethylrhodamine ester (TMRE, Invitrogen). Glucose consumption was evaluated using 2-NBDG (Molecular Probes). 2-NBDG is a derivative of glucose modified with a fluorescent amino group at the C-2 position. The C-2 position is the same position that the ^18^F atom is bound to in FDG. The 2-NBDG is uptaken into the cells and then decomposed into non-fluorescent forms^27^.

For these assays, 0.75 × 10^5^ cells were grown. After addition of the fluorophores (30 µM H_2_DCFDA, 100 nM TMRE, 100 µM 2-NBDG) and incubation at 37 °C for 30 min in the dark, cells were collected in DMEM and analyzed immediately with a MACSQuant® (Miltenyi-Biotec) flow cytometer. Forward and side scatter were used to gate the viable population of cells, and the mean fluorescence intensity was determined with FLOWJO software (TreeStar). Assays were performed by duplicate in at least 3 independent experiments.

### Statistics

All the statistics were carried out using the SPSS software version 15.0. The relationship between the cumulative probability of OS, PFS and analyzed predictors, was calculated with the Kaplan-Meier method. The population was divided based on the median or tercile. The medium and high terciles for GAPDH/MTCO1 ratio were grouped since they behaved in a similar way.

Bivariate correlations studies between PGC-1alpha, GAPDH/MT-CO1, and mitosis number in patient studies were analyzed using the Pearson correlation test. Differences in SUVmax levels between GAPDH/MT-CO1 ratio groups were evaluated using ANOVA.

ANOVA followed by Bonferroni post-test for multiple comparisons were used to analyze the differences in clonogenicity, migration, galactose to glucose growth ratios, oxygen consumption, ROS levels, glucose consumption and PGC-1alpha or GAPDH/MT-CO1 ratio mRNA levels among the cell lines. 2-tailed Student’s t test was used to analyze differences in MIMP, cell viability and clonogenicity after metformin treatment. In all cases, values of P < 0.05 were considered statistically significant.

### Data Availability

The datasets generated during the current study are available from the corresponding author on reasonable request.

## References

[1] Vansteenkiste J (2013). Early and locally advanced non-small-cell lung cancer (NSCLC): ESMO Clinical Practice Guidelines for diagnosis, treatment and follow-up. Ann. Oncol..

[2] Novello S (2016). Metastatic non-small-cell lung cancer: ESMO Clinical Practice Guidelines for diagnosis, treatment and follow-up. Ann. Oncol..

[3] Garrido P (2007). Long-term survival associated with complete resection after induction chemotherapy in stage IIIA (N2) and IIIB (T4N0-1) non-small-cell lung cancer patients: The Spanish Lung Cancer Group trial 9901. J. Clin. Oncol..

[4] Palka M (2016). Cisplatin plus vinorelbine as induction treatment in stage IIIA non-small cell lung cancer. Oncol. Lett..

[5] Xian Guo S, Jian Y, Chen YL, Cai Y, Zhang QY (2016). Neoadjuvant Chemoradiotherapy vesus Chemotherapy alone Followed by Surgery for Resectable Stage III Non-Small-Cell Lung Cancer: a Meta-Analysis &amp; Fang fang Tou Materials and Methods. Nat. Publ. Gr..

[6] Custodio A, Méndez M, Provencio M (2012). Targeted therapies for advanced non-small-cell lung cancer: Current status and future implications. Cancer Treatment Reviews.

[7] Rusch V (2016). Stage III Non-small Cell Lung Cancer. Semin. Respir. Crit. Care Med..

[8] Warburg O, Wind F, Negelein E (1927). The Metabolism of Tumors in the Body. J Gen Physiol.

[9] Heiden MV, Cantley L, Thompson C (2009). Understanding the Warburg effect: the metabolic requirements of cell proliferation. Science (80-.)..

[10] Andrzejewski, S. *et al*. Short Article PGC-1 a Promotes Breast Cancer Metastasis and Confers Bioenergetic Flexibility against Short Article PGC-1 a Promotes Breast Cancer Metastasis and Confers Bioenergetic Flexibility against Metabolic Drugs. 1–10 (2017). 10.1016/j.cmet.2017.09.006. 10.1016/j.cmet.2017.09.006 28988825

[11] Sancho P (2015). MYC/PGC-1α balance determines the metabolic phenotype and plasticity of pancreatic cancer stem cells. Cell Metab..

[12] LeBleu, V. S. *et al*. PGC-1α mediates mitochondrial biogenesis and oxidative phosphorylation in cancer cells to promote metastasis. *Nat*. *Cell Biol*. **16**, (2014). 10.1038/ncb3039 PMC4369153 25241037

[13] Girnun GD (2012). The diverse role of the PPARγ coactivator 1 family of transcriptional coactivators in cancer. Semin. Cell Dev. Biol..

[14] Tan Z (2016). The Role of PGC1 in Cancer Metabolism and its Therapeutic Implications. Mol. Cancer Ther..

[15] Teicher Ba, Linehan WM, Helman LJ (2012). Targeting cancer metabolism. Clin. Cancer Res..

[16] Zhao Y, Butler EB, Tan M (2013). Targeting cellular metabolism to improve cancer therapeutics. Cell Death Dis..

[17] Sborov DW, Haverkos BM, Harris PJ (2015). Investigational cancer drugs targeting cell metabolism in clinical development. Expert Opin Investig Drugs.

[18] Urvay SE, Yucel B, Erdis E, Turan N (2016). Prognostic Factors in Stage III Non-Small-Cell Lung Cancer Patients. Asian Pac. J. Cancer Prev..

[19] Cuezva JM (2004). The bioenergetic signature of lung adenocarcinomas is a molecular marker of cancer diagnosis and prognosis. Carcinogenesis.

[20] Vallejo CG (2013). Evaluation of mitochondrial function and metabolic reprogramming during tumor progression in a cell model of skin carcinogenesis. Biochimie.

[21] Bridges HR, Jones AJY, Pollak MN, Hirst J (2014). Effects of metformin and other biguanides on oxidative phosphorylation in mitochondria. Biochem. J..

[22] Appleby RD (1999). Quantitation and origin of the mitochondrial membrane potential in human cells lacking mitochondrial DNA. Eur. J. Biochem..

[23] Kadota K (2012). A grading system combining architectural features and mitotic count predicts recurrence in stage I lung adenocarcinoma. Mod. Pathol..

[24] Søes S (2013). Identification of accurate reference genes for RT-qPCR analysis of formalin-fixed paraffin-embedded tissue from primary non-small cell lung cancers and brain and lymph node metastases. Lung Cancer.

[25] Cruz-Bermúdez A (2015). Enhanced tumorigenicity by mitochondrial DNA mild mutations. Oncotarget.

[26] Cruz-Bermúdez A (2016). Functional Characterization of Three Concomitant MtDNA LHON Mutations Shows No Synergistic Effect on Mitochondrial Activity. PLoS One.

[27] Zou Chenhui, Wang Yajie, Shen Zhufang (2005). 2-NBDG as a fluorescent indicator for direct glucose uptake measurement. Journal of Biochemical and Biophysical Methods.

